# Synbiotics Supplements Lower the Risk of Hand, Foot, and Mouth Disease in Children, Potentially by Providing Resistance to Gut Microbiota Dysbiosis

**DOI:** 10.3389/fcimb.2021.729756

**Published:** 2021-09-30

**Authors:** Xiaoying Guo, Zixin Lan, Yaling Wen, Chanjiao Zheng, Zuhua Rong, Tao Liu, Siyi Chen, Xingfen Yang, Huimin Zheng, Wei Wu

**Affiliations:** ^1^ Guangdong Provincial Institute of Public Health, Guangdong Provincial Center for Disease Control and Prevention, Guangzhou, China; ^2^ School of Public Health, Southern Medical University, Guangzhou, China; ^3^ The Second Clinical Medical College, Southern Medical University, Guangzhou, China; ^4^ School of Mathematics and Computational Science, Guilin University of Electronic Technology, Guangxi, China; ^5^ Modern Service Industry Department, Guangzhou Technician College, Guangzhou, China

**Keywords:** hand, foot, and mouth disease, gut microbiota, synbiotics, probiotics, prebiotics

## Abstract

**Background:**

Hand, foot and mouth disease (HFMD) is an acute enterovirus-induced disease. Gut microbiota dysbiosis has been identified as a factor that plays an important role in enteral virus infection, but the gut microbiota profile in hand, foot and mouth disease has rarely been studied in a large population.

**Methods:**

A total of 749 children (HFMD: n = 262, healthy control: n = 487) aged 2 to 7 years were recruited from hospitals and communities in the period from May to July, 2017. Clinical and demographical information was collected by trained personnel, and fecal samples were collected and processed for 16S ribosomal RNA(rRNA) gene sequencing.

**Results:**

We observed a significant alteration in the microbiota profile of children with HFMD compared with that of control children. Patients with enteroviruses A71(EV71) positive had more dysbiotic gut microbiota than those with coxsackievirus A16 (CAV16) positive. We found that *Prevotella* and *Streptococcus* were enriched in children with HFMD, whereas beneficial bacteria, including *Bifidobacterium* and *Faecalibacterium*, were depleted. Children with synbiotics supplements had lower risk of HFMD and we observed that the gut microbiota of HFMD patients who were administered synbiotics exhibited potential resistance to the dysbiosis detected in HFMD.

**Conclusions:**

This study suggested that the gut microbiota of patients with hand, foot and mouth disease exhibits dysbiosis and that synbiotics supplements potentially helps maintain the homeostasis of the gut flora.

## Introduction

Hand, foot, and mouth disease (HFMD), a gastrointestinal contagious disease that mostly affects individuals under the age of 10 years, is one of the top three class C notifiable infectious diseases in China (Ying et al., 2019). Hence, HFMD poses a great threat to pediatric and neonatal populations in Asian countries.

The HFMD pathogens, mainly enteroviruses A71(EV71) and coxsackievirus A16 (CVA16), target the gastrointestinal (GI) epithelium and utilize intestinal bacteria to enhance multiplication, pathogenesis, and transmission and thereby lead to development of the disease. Additionally, the gut microbiota has been reported to work in conjunction with the intestinal barrier to orchestrate a defense network that impedes the invasion of pathogens and maintains the homeostasis and functionality of the gut (Sanz and De, 2009; Odenwald and Turner, 2017; Takiishi et al., 2017; Robinson, 2019). Dysbiosis of the gut microbiota could also lead to intestinal barrier disruption by influencing the self-renewal of epithelial cell and the secretion of the mucus layer and tight junctions of intestinal epithelial cells, which could increase the susceptibility of the host to immune or inflammatory disorders, including enterovirus infection (Wu et al., 2013; Winglee et al., 2014; Jakobsson et al., 2015; Schuijt et al., 2015; Sun et al., 2016; Heidrich et al., 2018; Alam and Neish, 2018). While majority studies had primarily focused on patients in epidemiological and virological studies, the role of the fecal bacterial microbiome in HFMD progression remains barely undocumented. Thus far, only limited studies with very small sample sizes have suggested the existence of gut microbiota dysbiosis in children with HFMD, and larger population studies are urgently needed to reveal the relationship and the underlying mechanisms. Probiotics and prebiotics are widely utilized as complements to nutrition strategies designed to reinforce intestinal immunity. Several studies have provided scientific evidence of the benefits resulting from the use of probiotics and prebiotics for intestinal diseases. Currently, researchers are considering the use of probiotics and prebiotics for the prevention of HFMD, but no related studies have been conducted to date. It is necessary to assess whether the use of probiotics and prebiotics exhibits potential preventive efficacy on intestinal flora in children with HFMD.

Here, we conducted a two-site recruitment and investigated 749 participants (including 262 HFMD patients and 487 healthy children) from two regions in Guangdong, China. By collecting their information and fecal samples, we aimed to explore the differences in gut microbiota communities between children with HFMD and healthy children and the potential effects of probiotics and prebiotics on HFMD infection.

## Materials and Methods

### Study Design and Subjects

All study participants were recruited from an investigation that aimed to investigate the distribution of intestinal bacteria and its influencing factors in children with HFMD that was conducted in Guangdong Province, from May to July, 2017. The investigation sites were Xinhui District of Jiangmen City and Longchuan County of Heyuan City, Guangdong Province. Two local hospitals, including a children’s hospital and a county-level hospital, were selected in each investigation site. HFMD patients were recruited from local hospitals, and healthy children were recruited from the same hospitals or nearby communities where the patients live. The inclusion criteria were as follows: a) 2- to 7-year-old children; b) living in the county or district for at least 6 months. The exclusion criteria: a) having a critical illness, herpetic pharyngitis and severe organic lesions; b) having a disease related to the risk factors for the study, including diarrhea, gastroenteritis, previous diagnosis of HFMD, and herpetic angina, among others; and c) taking antibiotics within one month. The HFMD cases were diagnosed by medical institutions and clinics in the county or district according to the Chinese Guidelines for HFMD diagnosis and treatment (2010 Edition) issued by the Ministry of Health^1^ 1Ministry of Health of the People’s Republic of China.(2010). Diagnosis and treatment guideline on hand-foot-mouth disease. http://yzs.satcm.gov.cn/gongzuodongtai/2018-03-24/3073.html. [Accessed March 1, 2017].. The study design is illustrated in **Supplementary Figure 1**. The participants were required to complete a questionnaire including demographic characteristics, hygienic habits, and supplements information and medication usage within one month. In total, 262 patients with HFMD and 487 healthy children were recruited in our study. Before any investigations or data gathering, each legal guardian of admitted participant signed a written informed consent form. This study was approved by the Review Board of Guangdong Provincial Centers for Disease Control and Prevention.

### Samples and Sequencing

Fecal samples were collected from all 749 subjects who completed the questionnaire, and within 2 h, these samples were placed in a refrigerator at 4°C for temporary preservation. Within 24 h, the samples were stored at -80°C before analysis.

According to the instructions provided with the QIAamp DNA Stool Mini kit (QIAGEN, Hilden, Germany), total DNA were extracted from fecal samples. The V4 region of 16S rRNA gene was amplified by polymerase chain reaction (PCR) from the total isolated DNA samples. The amplification reaction conditions were as follows: initial activation step of 94°C for 2 min followed by 30 cycles of 94°C for 30 s, 52°C for 30 s and 72°C for 30 s and a final incubation at 72°C for 5 min. The V4 region of 16S rRNA was sequenced by second-generation sequencing (Illumina HiSeq 2000) in combination with third-generation sequencing (PacBio SMRT).

### Bioinformatic Analysis

Using R V.4.0.3 (under RStudio V. 1.4.1106), the 16S rRNA gene reads were merged, subjected to quality control and clustered into amplicon sequence variants (ASVs). The R package DADA2 was used to process the Illumina demultiplexed paired-end sequenced dataset to correct for amplicon errors, to identify chimeras and to merge paired-end reads. Low-quality regions at the tails in the sequences were first trimmed for removal. Based on the estimated error rate, filtered forward and reverse sequences were then dereplicated. Unique sequences were identified using pseudo-pooling, and paired reads were merged to generate fully denoised sequences. The denoised sequences were then assigned to ASVs. Taxonomically, the samples were classified according to the Greengenes reference database (McDonald et al., 2012).

The values for alpha diversity (Chao1 index, Shannon’s index, PD whole tree index and observed ASVs), beta diversity (Bray Curtis, weighted and unweighted UniFrac distance metrics) and principal coordinate analysis (PCoA) were generated with R. Permutational multivariate analysis of variance was performed to determine whether the compositions of the microbiota differed between groups. To identify the enriched and depleted bacteria between groups, differential taxa in the cases and the controls were discovered by LEfSe using the web-based program Galaxy (Segata et al., 2011).

### Statistical Analysis

The categorical variables are expressed as means and standard deviation and the numeric variables are reported as percentages. The characteristics of the individuals in the cohort were compared by Student’s *t*-test and Chi-squared test. The Wilcoxon rank-sum test was used to compare the Chao1, Shannon index, observed ASVs, and PD whole-tree index between the cases and controls. The relative abundances of genera were compared among the groups using the Kruskal-Wallis rank sum test. Benjamini-Hochberg method was used to adjust for multiple comparation. We used logistic regression models to calculate the odds ratios between the HFMD and control groups, and the Wald’s test and likelihood ratio test were then used to analyze the relationship of the groups. Propensity Score Matching (PSM) was applied to minimize the effect of potentially confounding factors, such as matching two cohorts to have the same distribution of age and sex. Then, a propensity score for the relative abundances of selected genera of each individual was calculated after using results of this model. Finally, matching with a 1:1 ratio was performed using a nearest-neighbor approach with caliper restriction between case group and controls. In addition, the association between genus and HFMD was assessed by multivariate association with linear models 2 (MaAsLin2) as described by Morgan et al. (Himel, 2021). Age and sex were used as confounders, and the false discovery rate was limited to 0.05. All analyses were performed using R software, version 4.0.2 (R Foundation for Statistical Computing, Vienna, Austria). Statistical significance was accepted at P < 0.05.

## Results

### Study Population

In the present study, a total of 749 children (487 healthy childrenand 262 children with HFMD) from Heyuan and Jiangmen were included. The mean age of the patients with HFMD was 3.6 years, whereas the average age of the control group was 4.6 years. Males made up 53 percent of the subjects in the control group, whereas 62 percent of the group of children with HFMD were males. The age, sex, probiotics, prebiotics and synbiotics supplements, hygienic habits, city information and statistics and of the study participants are detailed in **Table 1**.

**Table 1:** Demographic characteristics and probiotics, prebiotics, and synbiotics supplement of the participants.

	Control (N = 487)	HFM (N = 262)	*P*-value
**Age** ^**a**^			
Mean ( ± SD)	4.6 (± 1.2)	3.6 (± 1.3)	<0.001^*^
**Sex** ^**b**^			
Male	256 (53%)	162 (62%)	0.0184^*^
**Supplements type** ^**b**^			
No supplements	116 (24%)	64 (24%)	<0.001^*^
Pre	8 (2%)	8 (3%)	
Pro	214 (44%)	158 (60%)	
Syn	149 (31%)	32 (12%)	
**City** ^**b**^			
Heyuan	239 (49%)	192 (73%)	<0.001^*^
Jiangmen	248 (51%)	70 (27%)	
**Wash hands after playing outside** ^**b**^			
Yes	486 (100%)	239 (91%)	<0.001^*^
No	1 (0%)	23 (9%)	
**Suck fingers** ^**b**^			
Yes	196 (40%)	134 (51%)	<0.001^*^
No	291 (60%)	128 (49%)	
**Roll and play on the ground** ^**b**^			
Yes	324 (66%)	222 (85%)	<0.001^*^
No	163 (34%)	40 (15%)	
**Roll and play on the ground** ^**b**^			
Yes	432 (88%)	241 (92%)	0.15
No	55 (12%)	21 (8%)	

Pro, probiotics supplements.

Pre, prebiotics supplements.

Syn, synbiotics supplements.

a Student’s t-test.

b Chi-squared test.

*Significant differences with P-value < 0.05.

### Altered Gut Microbiota Profile of Children With HFMD

**Figure 1:**
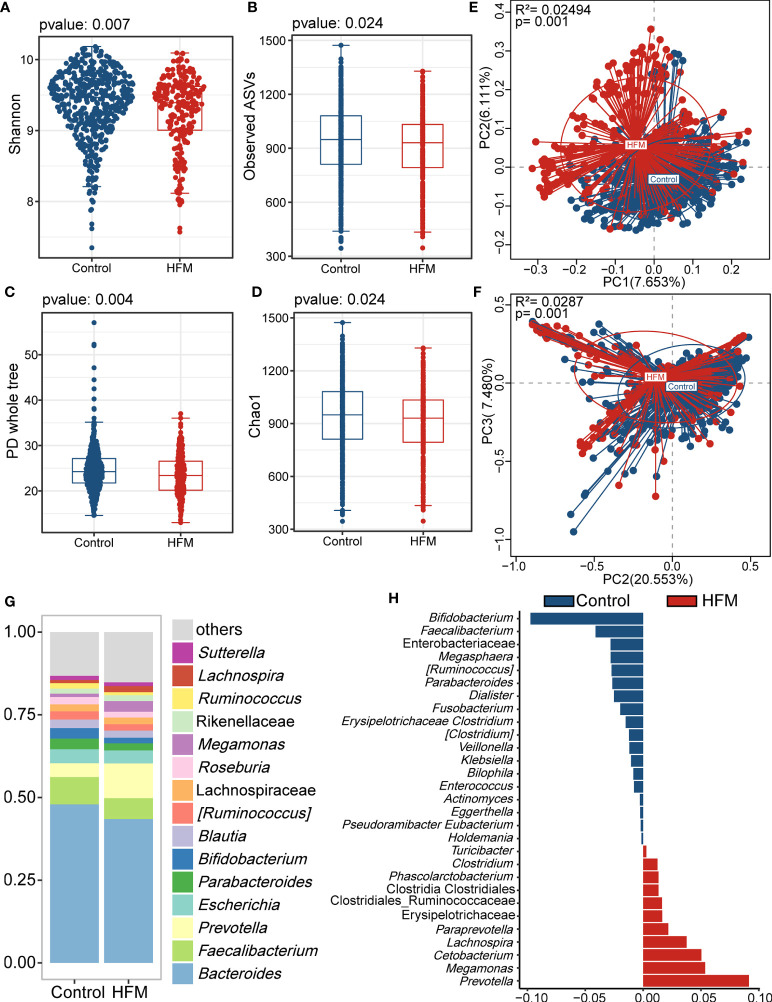
Altered bacterial microbiota biodiversity and composition in HFMD based on whole cohort. **(A–D)** Alpha diversity. Comparison of alpha-diversity indices (Shannon, observed ASVs, PD whole tree and Chao 1 index) between the male HFMD and male control groups (Wilcoxon rank-sum tests). **(E, F)** Principal coordinate analysis based on unweighted and unweighted UniFrac distances revealed that the bacterial communities of patients with HFMD clustered separately from the bacterial communities of healthy control children. Each point represents a single sample, which is colored based on the group. PC1, PC2 and PC3 represent the top three principal coordinates that captured most of the diversity. The explanation of diversity captured by the coordinate is given as a percentage. **(G)** Relative abundance of the microbiota at genus level. **(H)** Differences in the bacterial taxon between patients with HMFD and control children. The beta coefficient was calculated with the multivariate association with linear models 2 (MaAsLin2) after adjusting for age and sex.

The gut microbiota analysis revealed significant differences in the alpha and beta diversity. The HFMD group exhibited significantly lower alpha diversity indices, including the Shannon index, observed ASVs, PD whole-tree index, and Chao1 index, than the control group. This finding suggested that the gut microbiome of the HFMD group exhibited lower richness and reduced evenness than that of the control group (**Figures 1A–D**). According to the PCoA plot based on the unweighted and weighted UniFrac distances, the gut microbiota of the HFMD group differed significantly from that of the control group, which indicated that the composition of the gut microbiota of the HFMD group was significantly different from that of the control group (**Figures 1E, F**).

Analysis of the bacterial genera stack plots of the HFMD and control groups revealed that *Faecalibacterium*, *Prevotella*, *Bacteroidetes*, *Escherichia*, *Parabacteroides* and *Bifidobacterium* were the major genera in both groups (**Figure 1G**). To further identify the bacteria that differed between the HFMD and control groups, we performed a MaAsLin2 analysis using age and sex as confounders and identified genera that exhibited significant differences (**Figure 1H**). *Prevotella*, *Cetobacterium*, and *Megamonas* were enriched in the feces of patients with HFMD compared with those of the control group, whereas *Bifidobacterium* and *Faecalibacterium* were depleted in patients with HFMD.

**Figure 2:**
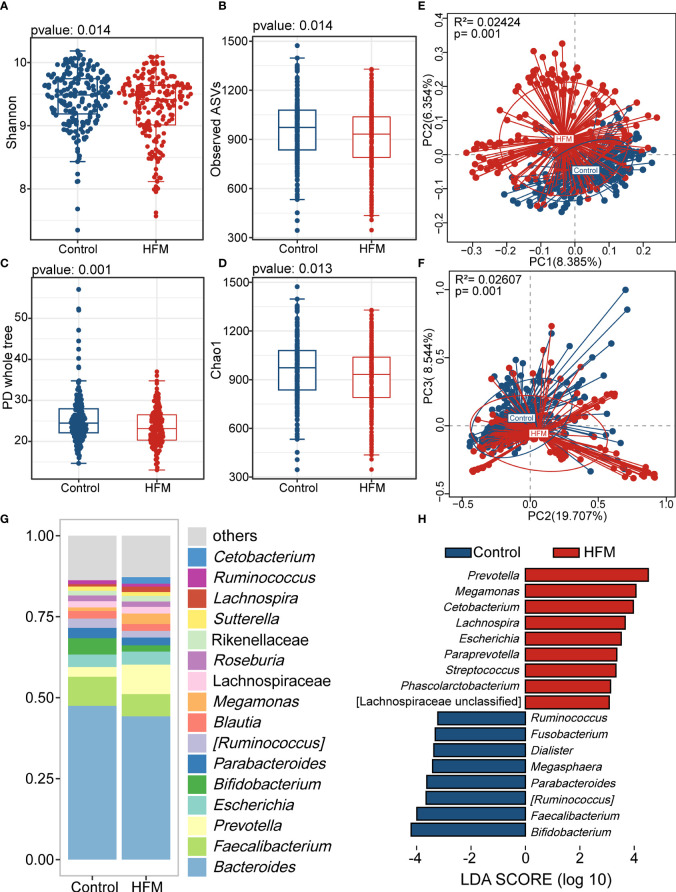
Altered bacterial microbiota biodiversity and composition in HFMD based on cohort matched age and sex. **(A–D)** Alpha diversity. Comparison of alpha-diversity indices (Shannon, observed ASVs, PD whole tree and Chao 1 index) between the HFMD and control groups (Wilcoxon rank-sum tests). **(E, F)** Principal coordinate analysis based on unweighted and weighted UniFrac distances revealed that the bacterial communities of patients with HFMD clustered separately from the bacterial communities of healthy control children. Each point represents a single sample, which is colored based on the group. PC1, PC2 and PC3 represent the top three principal coordinates that captured most of the diversity. The explanation of diversity captured by the coordinate is given as a percentage. **(G)** Relative abundance of the bacterial microbiota at the genus level. **(H)** Differences in the bacterial taxon abundance between patients with HMFD and control children by using Linear discriminant analysis effect size (LEfSe) analysis. HFMD-enriched taxa are indicated with a positive LDA score, and taxa enriched in healthy controls have a negative score. Only taxa meeting an LDA significance threshold of >3 are shown. LDA, linear discriminant analysis.

Previous studies have revealed that age and sex should be considered co-factors in analysis of the gut microbiota (Xiu et al., 2021). Thus, we employed Propensity Score Matching (PSM) to adjust for age and sex in our study population. After PSM, the mean age of two cohorts was 4.0 ± 1.2 years. The majority of subjects (63%) were male. There were significant differences in various supplement types between matched groups. In the hygienic habits analysis, washing hands after playing outside, sucking fingers and rolling and playing on the ground presented significantly difference between two groups (**Supplementary Table 1**). In the adjusted cohort, the alpha-diversity indices of the HFMD group were significantly lower than those of the control group (**Figures 2A–D**). The PCoA plot also showed that the gut flora of the HFMD group was different from that of the control group (**Figures 2E, F**). The majority of genera in this adjusted cohort were in consistence with the previous results (**Figure 2G**). We then performed the linear discriminant analysis effect size (LEfSe) analysis and identified genera that exhibited significant differences, including *Prevotella*, *Cetobacterium*, *Megamonas*, *Bifidobacterium* and *Faecalibacterium* (**Figure 2H**).

**Figure 3:**
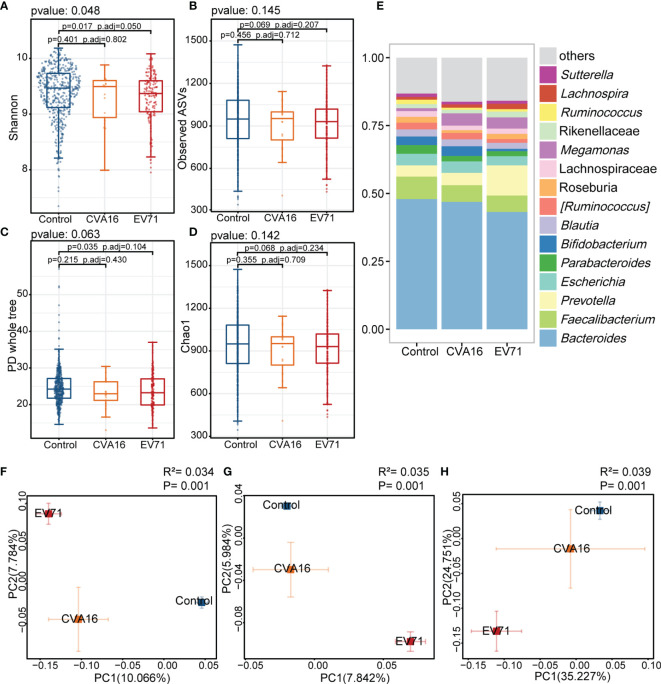
Comparison of the gut microbiota between EV71, CVA16, and control group. **(A–D)** Alpha diversity. Comparison of alpha-diversity indices (Shannon, observed ASVs, PD whole tree and Chao 1 index) among EV71-infected HFMD, CVA16-infected HFMD and control groups (Kruskal-Wallis tests). **(E)** Relative abundance of the bacterial microbiota at the genus level among EV71, CVA16, and control group. **(F–H)** Beta diversity. Principal coordinate analysis (PCoA) based on the Bray-Curtis distance **(F)**, unweighted UniFrac distances **(G)** and weighted UniFrac distances **(H)** revealed that the relationship of bacterial communities among EV71-infected HFMD, CVA16-infected HFMD and control groups. The explanation of diversity captured by the coordinate is given as a percentage.

In the present study, there were 144 patients infected EV71 while 21 patients infected CVA16. Shannon index of EV71 group was lower than control group and PCoA analysis showed a longer distance of EV71 to control group comparing with CVA16 (**Figure 3**). In the analysis within each sex group, the results echoed our results mentioned above. The PD whole tree index was lower in HFMD group comparing with control group and both showed significant gut microbiome compositional shift. *Prevotella*, *Cetobacterium* and *Megamonas* were enriched while *Faecalibacterium* and *Bifidobacterium* were depleted in HFMD groups in both male and female group (**Supplementary Figures 2, 3**).

These results showed that the gut microbiota was altered in children with HFMD, which indicated that the gut microbiota were associated with hand, foot, and mouth disease.

### Synbiotics Supplements Exerted a Protective Effect in HFMD

Probiotics and prebiotics supplements have been demonstrated to play positive roles in the modulation of the gut microbiota (Ki et al., 2012; Liu et al., 2015). To examine whether probiotics, prebiotics and synbiotics supplements exerted a protective effect on HFMD, we performed logistic regression models to calculate the odds ratios of HFMD for probiotics, prebiotics and synbiotics supplement use. After adjusting for age and sex, the odds ratios (ORs) (95% CIs) for HFMD according to the application of probiotics, prebiotics and synbiotics were 1.65 (0.55,4.92), 1.59 (1.07,2.37) and 0.49 (0.29,0.82), respectively (**Supplementary Table 2**). In PSM-adjusted cohort, the ORs of all groups (95% CIs) were reduced to 1.34 (0.39, 4.65), 1.8 (1.12, 2.9) and 0.45 (0.25, 0.81), respectively (**Supplementary Table 3**). We further adjusted for age, sex and hygiene, and consistent findings were observed in **Table 2**. The results indicated that children who utilized synbiotics were at lower risk of infection than those who used probiotics alone or prebiotics alone.

**Table 2:** Effects of the intake of probiotics, prebiotics, or synbiotics on gut microbiota of patients with HFMD after adjusting for age, sex and hygiene.

	Crude OR (95 % CI)	Adjusted OR (95 % CI)	*P* (Wald test)	*P* (LR test)
No supplements	Reference	Reference		
Pre	1.81 (0.65,5.06)	1.35 (0.41,4.40)	0.619	
Pro	1.34 (0.93,1.93)	1.70 (1.12,2.58)	0.013^*^	
Syn	0.39 (0.24,0.63)	0.57 (0.33,0.98)	0.044^*^	
Age	0.53 (0.47,0.61)	0.54 (0.47,0.62)	<0.001^*^	<0.001^*^
Sex (male)	1.46 (1.08,1.99)	1.44 (1.01,2.04)	0.042^*^	0.041^*^
Wash hands after playing outside	0.02 (0,0.16)	0.02 (0,0.15)	<0.001^*^	<0.001^*^
Suck fingers	1.55 (1.15,2.10)	0.90 (0.63,1.28)	0.545	0.545
Roll and play on the ground	2.79 (1.90,4.11)	2.00 (1.28,3.11)	0.002^*^	0.002^*^
Public toys	1.46 (0.86,2.47)	1.67 (0.90,3.10)	0.104	0.097

Pro, probiotics supplements.

Pre, prebiotics supplements.

Syn, synbiotics supplements.

95% CI, 95% confidence interval.

LR-test, likelihood ratio test.

*Significant differences with P-value < 0.05.

### Effect of Probiotics, Prebiotics and Synbiotics on the Gut Microbiota of Children With HFMD

**Figure 4:**
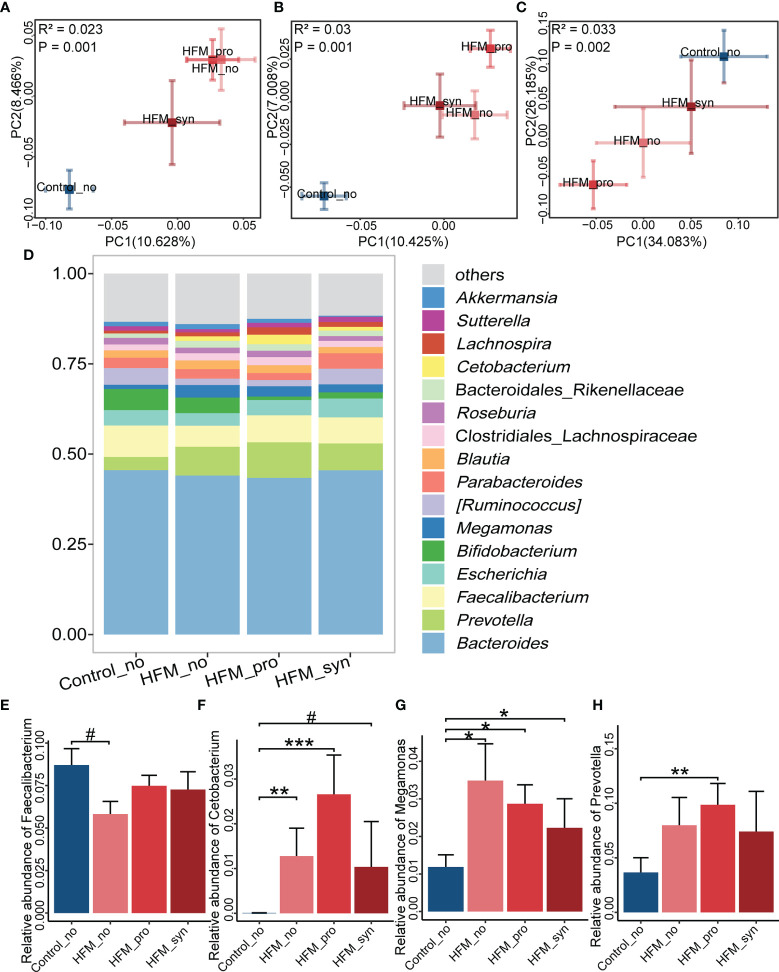
Comparison of the gut microbiota among the probiotics (Pro), prebiotics (Pre) and Synbiotics (Syn) supplements. **(A–C)** Beta diversity. Principal coordinate analysis (PCoA) based on the Bray-Curtis distance **(A)**, unweighted UniFrac distances **(B)** and weighted UniFrac distances **(C)** revealed the relationship of bacterial communities among the Pro, Pre and Syn groups. The explanation of diversity captured by the coordinate is given as a percentage. **(D)** Relative abundance at the genus level of the different groups. **(E–H)** Relative abundances of *Faecalibacterium*, *Cetobacterium*, *Megamonas* and *Prevotella* between various groups (Kruskal-Wallis rank sum test and multiple comparison between groups adjusted by Benjamini-Hochberg method). * indicates *P* < 0.05, ** indicates *P* < 0.01, *** indicates *P* < 0.001, ^#^ indicates *P* < 0.1.

In the studied cohort, the number of patients who received prebiotics was relatively small (n=8), and we thus had to perform an analysis of the gut microbiota without considering the population administered prebiotics alone. The PCoA plot showed that the gut microbiota structure of the patients who used synbiotics supplements was closer to that of the control group than that of patients with probiotics alone supplements and patients without supplement groups, as could be observed with different distance algorithms (**Figures 4A–C**). Most of the genera found in each group were similar, and the main genera were *Faecalibacterium*, *Prevotella*, *Bacteroidetes*, *Escherichia*, *Parabacteroides* and *Bifidobacterium* (**Figure 4D**). The abundance of genera identified as significantly altered in HFMD showed less extensive changes in the patients who were administered synbiotics. *Faecalibacterium* was depleted in patients who were not administered any supplement and was restored in patients applied synbiotics supplements (**Figure 4E**). *Cetobacterium*, *Megamonas* and *Prevotella* were increased in patients with probiotic supplements but tended to decrease in the patients applied synbiotics supplements (**Figures 4F–H**). These data suggested that the gut microbiota of the children who were administered synbiotics supplements showed potential resistance to HFMD-related dysbiosis.

## Discussion

HFMD has become an emerging public health problem in Asia, but there is currently no vaccine to protect against the responsible viruses. The present study aimed to seek answers to unanswered questions related to the gut microbiota. The differences in the gut microbiota patterns of patients with HFMD compared with those of the healthy group and the effect of synbiotics supplements on the intestinal flora of patients with HFMD were explored. We observed that the gut microbiota were altered in children with HFMD. Furthermore, children with HFMD who were administered synbiotics supplements exhibited reduced morbidity, and their gut microbiota showed potential resistance to HFMD-related gut flora dysbiosis.

First, the distribution characteristics of the gut microbiota of patients with HFMD differed from those of the gut microbiota of the healthy group. A reduction in bacterial diversity, which is considered to indicate an unhealthy state, has been observed in children with HFMD (Rivera-Chávez et al., 2016; Sassone-Corsi et al., 2016; Kriss et al., 2018). This result was consistent with previous research, which demonstrated that changes in the diversity of butyrate-producing bacteria and inflammation-inducing bacteria are associated with HFMD. In the present study, several bacteria were found to differ between the HFMD and control groups. Our results showed that *Prevotella* and *Streptococcus* were found at higher relative abundances in patients with HFMD than in the healthy group. *Prevotella* is widely considered a commensal genus, and a series of controversial reports on its pathogenicity have been published (Brook, 1998; Brook, 2004; Nagy, 2010). For example, *Prevotella copri*, a widely distributed species, has been linked to inflammatory diseases and intestinal barrier dysfunction by directly promoting the upregulation of inflammatory cytokines. Additionally, *Prevotella* is a lipopolysaccharide-producing anaerobic gram-negative bacterium that can cause inflammatory reactions and damage the gut barrier (Hofstad et al., 1993; Choi et al., 2011). *Streptococcus*, which is one of the most classic opportunistic pathogens, comprises a wide variety of pathogenic bacterial strains. *Streptococcus* is one of the most prevalent inhabitants of the respiratory tract (Engholm et al., 2017), skin surface (Ho et al., 2021) and gut (Sokol et al., 2017; Ooi et al., 2018) of adults and children. Studies have shown that influenza virus can interact with *S. pneumoniae* and induce susceptibility to viruses by activating cytokines and dendritic cells (Short et al., 2012). The enrichment of *Streptococcus* has also been observed in the gut microbiota of patients with viral hepatitis cirrhosis (Wang et al., 2019). The cause of HFMD is enteral virus infection, e.g., infection with EV71 and CVA16. A study found that EV71 is transported from the intestine to peripheral tissue and other organs, which suggests that the small intestine is a gateway for EV71 infection *in vivo* (Feng et al., 2020). EV71 infection could reduce the expression of goblet cell-derived mucins (Good et al., 2019), which indicates that EV71 infection could alter the luminal environment of the gut microbiota. Previous data have confirmed that EV71 infect and replicate in intestinal epithelial cells by activating the p38 mitogen-activated protein kinase (MAPK) and protein kinase signaling pathways. This inflammatory pathway overlaps with the inflammatory reactions caused by bacterial lipopolysaccharide or translocation. In addition, EV71 is most frequently associated with severe diseases (Shu et al., 2015). In our study, the gut microbiota of patients infected with EV71 were more dysbiotic than that of patients with CVA16. Hence, in HFMD pathogenesis, the observed gut dysbiosis might interact with enteroviruses.

In contrast, the relative abundances of *Faecalibacterium* and *Bifidobacterium* were decreased in the feces of the HFMD group. These two bacterial genera, which are generally considered producers of short-chain fatty acids, are reportedly capable of regulating intestinal epithelial nutrition, stabilizing immune homeostasis and reinforcing intestinal barrier functions (Hiippala et al., 2018; Routy et al., 2018). *Bifidobacteria* protect gut mucus against diet-induced microbiota-mediated deterioration, and gut barrier defects (Schroeder et al., 2018). *Bifidobacteria* have also been identified as health-promoting genera that increases butyrate production and thus contributes to improvements in the gut barrier and metabolic outcomes (Seganfredo et al., 2017). Similarly, *Faecalibacterium* exhibits an enriched polyphenol-rich dietary pattern, which could improve gut barrier function (Del Bo' et al., 2020), and the prophylactic use of *Faecalibacterium* could also prevent the acute breakdown of the colonic epithelial barrier (Lapiere et al., 2020). Thus, in both the research and healthcare product markets, *Bifidobacterium* is a very common commercialized probiotics, and *Faecalibacterium* shows strong promise as effective and safe probiotics.

In clinical practice, probiotics and prebiotics are widely used as supplements for healthy children or are included in therapeutic approaches for many diseases (Krumbeck et al., 2016). In this study, we found that children who were administered supplements exhibited a lower risk of HFMD-related infection, whereas the application of probiotics or prebiotics alone was not found to be significantly associated with HFMD risk. This very interesting finding indicates that synbiotics was related to an improved protective effect. A series of studies conducted by Professor D. A. Mills from the University of California Davis revealed that *Bifidobacterium* can process the prebiotics in human milk oligosaccharides and that the colonization of the probiotics *Bifidobacterium breve* could also be influenced by prebiotics (Underwood et al., 2017). These studies suggested a potential close association between probiotics and prebiotics, and this association is also being investigated by Professor Mills’s group. The intake of prebiotics may modulate the composition of the microbiota, be fermented by the beneficial gut microbiota and increase the abundance of probiotics (Gibson and Roberfroid, 1995; Pandey et al., 2015). Synbiotics, defined as combinations of probiotics and prebiotics, could benefit the host by modulating the gut microbiota (Gibson and Roberfroid, 1995). Previous studies have reported that synbiotics exert protective or preventive effects against gastrointestinal or other diseases by maintaining the gut flora structure (Shimizu et al., 2018; Tian et al., 2018). Similarly, in our study, the beta diversity analysis showed that the composition of the gut flora of patients applied synbiotics was closer to that of the control group than to those of the other patient groups. The analysis of the abundances of identified biomarkers indicated their relative restoration and depletion in patients applied synbiotics compared with those found in the other patient groups. These gut microbiota alterations suggested that the gut microbiota of the children who received synbiotics supplements could acquire resistance to HFMD-related gut flora changes, which might manifest as a reduction in HFMD susceptibility.

Our study has some limitations. In the present study, we only collected information regarding whether the children were administered supplements, including probiotics and prebiotics. However, the specific types and dose of probiotics or prebiotics are unknown. Besides, prebiotics are also found in food (Roberta, et al., 2020), especially those that contain complex carbohydrates, such as fiber and resistant starch. Diet information wasn’t collected and dietary prebiotics assessment were required in further study. Another major limitation is that all the samples from the HFMD group were collected after infection. Thus, the analysis could only suggest a potential bacterial resistance in the children who were administered synbiotics. However, the existence of this resistance in the gut microbiota should be validated in future follow-up studies with repeated and larger populations sampled before and after infection.

To our knowledge, the present study involves the largest HFMD cohort used for gut microbiota analyses thus far. We found that the gut microbiota of children with HFMD exhibits dysbiosis, and synbiotics supplements might provide a potential protective effect against HFMD. However, further studies with a larger population and detailed information are needed to validate our findings and explore the underlying mechanisms of HFMD. In conclusion, our study indicated the changes in the composition of the gut flora of patients with HFMD and identified potential bacterial biomarkers of HFMD. Additionally, we provided new evidence showing that synbiotics might lower the susceptibility to HFMD by modulating the gut microbiota.

## Data Availability Statement

The datasets presented in this study can be found in online repositories. The names of the repository/repositories and accession number(s) can be found below: https://www.ebi.ac.uk/ena/browser/view/PRJEB45719.

## Ethics Statement

The studies involving human participants were reviewed and approved by Ethical Review Committee of Chinese Center for Disease Control and Prevention. Written informed consent to participate in this study was provided by the participants’ legal guardian/next of kin.

## Author Contributions

WW, HZ, and XY designed the study. ZR, CZ, TL, YW, and SC processed the samples. XG and ZL searched literature, analyzed the data, and drafted the manuscript. WW, HZ, and XY reviewed and revised the manuscript. All authors contributed to the article and approved the submitted version.

## Funding

This work was funded by the Science and Technology Planning Project Foundation of Guangzhou city (202102080584).

## Conflict of Interest

The authors declare that the research was conducted in the absence of any commercial or financial relationships that could be construed as a potential conflict of interest.

## Publisher’s Note

All claims expressed in this article are solely those of the authors and do not necessarily represent those of their affiliated organizations, or those of the publisher, the editors and the reviewers. Any product that may be evaluated in this article, or claim that may be made by its manufacturer, is not guaranteed or endorsed by the publisher.
