# Supplementary material for: Synbiotics Supplements Lower the Risk of Hand, Foot, and Mouth Disease in Children, Potentially by Providing Resistance to Gut Microbiota Dysbiosis
Source: Front Cell Infect Microbiol. 2021 Sep 30;11:729756. doi: 10.3389/fcimb.2021.729756 (PMC8515124; doi:10.3389/fcimb.2021.729756)
Supplement: Supplementary file 1 [file DataSheet_1.docx]

Supplementary Material

# Supplementary Figures and Tables

## Supplementary Figures


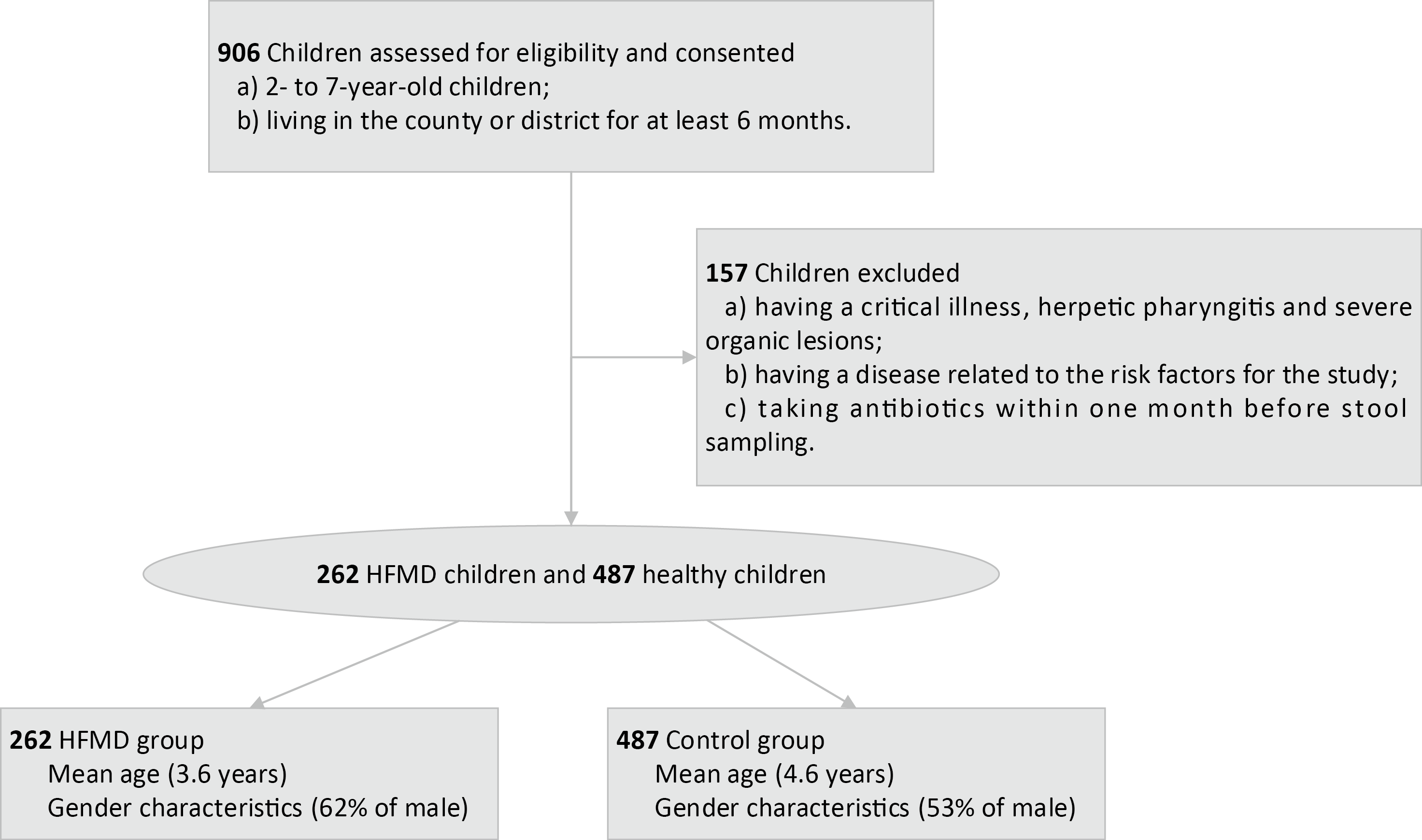


**Supplementary Figure 1.** The initial survey included 906 subjects who were assessed for eligibility and consented. After exclusion of health status and antibiotics usage history, 749 children were recruited, including 262 hand-foot-mouth disease children (HFMD) and 487 healthy children. In the HFMD group, the mean age of children was 3.6 years and males made up 53 percent of the group. In the control group, the average age of children was 4.6 years and 62 percent of the group of children


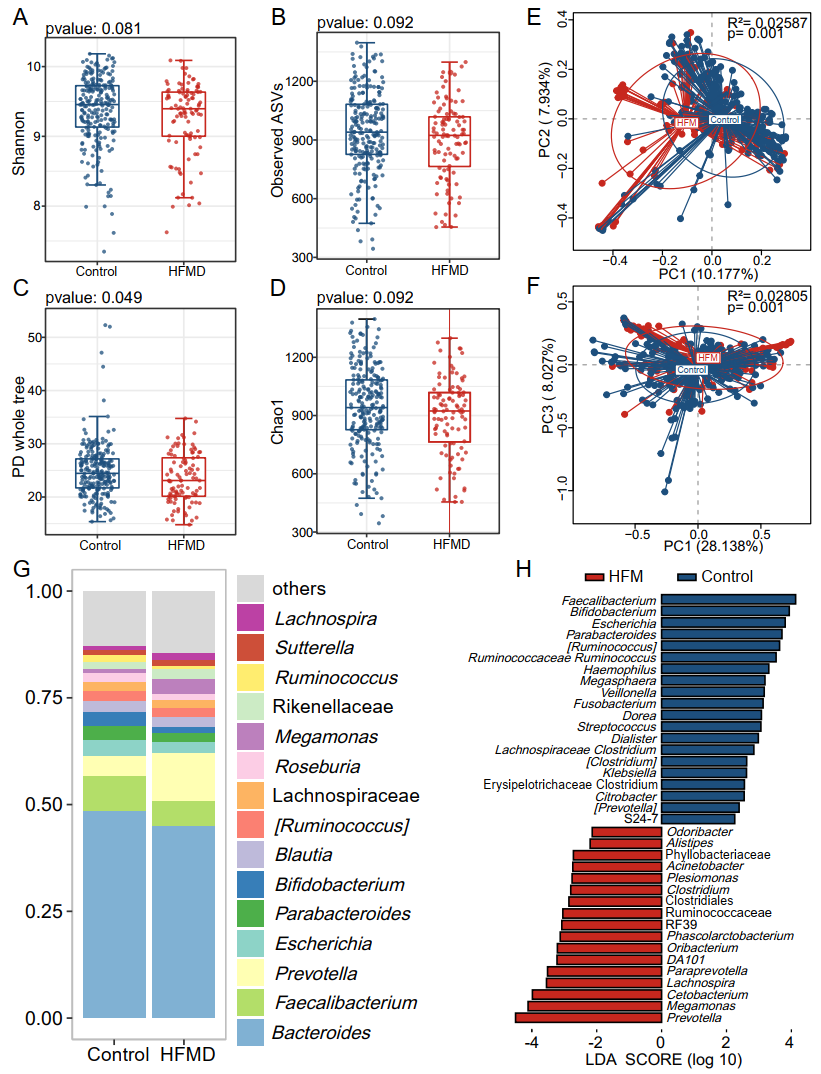


**Supplementary Figure 2.** Altered bacterial microbiota biodiversity and composition in female group. (A-D) Alpha diversity. Comparison of alpha-diversity indices (Shannon, observed ASVs, PD whole tree and Chao 1 index) between the female HFMD and female control groups (Wilcoxon rank-sum tests). (E, F) Beta diversity. Principal coordinate analysis based on weighted and unweighted UniFrac distances revealed that the bacterial communities of patients with HFMD clustered separately from the bacterial communities of healthy children. Each point represents a single sample, which is colored based on the group. PC1, PC2 and PC3 represent the top three principal coordinates that captured most of the diversity. The explanation of diversity captured by the coordinate is given as a percentage. (G) Relative abundance of the microbiota at genus level. (H) Differences in the bacterial taxon between patients with HMFD and healthy children by using Linear discriminant analysis effect size (LEfSe) analysis. HFMD-enriched taxa are indicated with a positive LDA score, and taxa enriched in healthy controls have a negative score. Only taxa meeting an LDA significance threshold of >2 are shown. LDA, linear discriminant analysis.


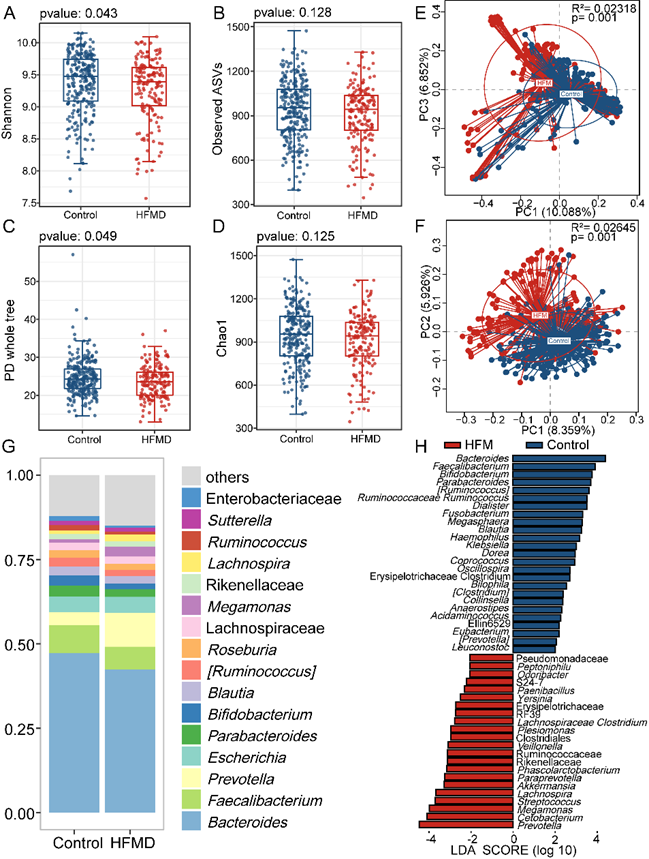


**Supplementary Figure 3.** Altered bacterial microbiota biodiversity and composition in male group. (A-D) Alpha diversity. Comparison of alpha-diversity indices (Shannon, observed ASVs, PD whole tree and Chao 1 index) between the female HFMD and female control groups (Wilcoxon rank-sum tests). (E, F) Beta diversity. Principal coordinate analysis based on weighted and unweighted UniFrac distances revealed that the bacterial communities of patients with HFMD clustered separately from the bacterial communities of healthy children. Each point represents a single sample, which is colored based on the group. PC1, PC2 and PC3 represent the top three principal coordinates that captured most of the diversity. The explanation of diversity captured by the coordinate is given as a percentage. (G) Relative abundance of the microbiota at genus level. (H) Differences in the bacterial taxon between patients with HMFD and healthy children by using Linear discriminant analysis effect size (LEfSe) analysis. HFMD-enriched taxa are indicated with a positive LDA score, and taxa enriched in healthy controls have a negative score. Only taxa meeting an LDA significance threshold of >2 are shown. LDA, linear discriminant analysis.

## Supplementary Tables

**Supplementary Table 1.** Characteristics of patients with HFMD and healthy children in Propensity Score Matching (PSM) adjusted cohort.

|  | **Control (N=203)** | **HFM (N=203)** | ***P*-value** |
| --- | --- | --- | --- |
| **Age** |  |  |  |
| Mean (±SD) | 4.0 (± 1.2) | 4.0 (± 1.2) | 1 |
| **Sex** |  |  |  |
| Female | 75 (37 %) | 75 (37 %) | 1 |
| Male | 128 (63 %) | 128 (63 %) |  |
| **Supplements type^a^** |  |  |  |
| No supplements | 58 (29 %) | 52 (26 %) | <0.001^*^ |
| Pre | 5 (2 %) | 6 (3 %) |  |
| Pro | 73 (36 %) | 118 (58 %) |  |
| Syn | 67 (33 %) | 27 (13 %) |  |
| **Wash hands after playing outside^a^** |  |  |  |
| Yes | 203(100%) | 185(91%) | <0.001^*^ |
| No | 0(0%) | 18(9%) |  |
| **Suck fingers^a^** |  |  |  |
| Yes | 84(41%) | 100(49%) | 0.11 |
| No | 119(59%) | 103(51%) |  |
| **Roll and play on the ground^a^** |  |  |  |
| Yes | 137(67%) | 169(83%) | <0.001^*^ |
| No | 66(33%) | 34(17%) |  |
| **Public toys^a^** |  |  |  |
| Yes | 176(87%) | 187(92%) | 0.08 |
| No | 27(13%) | 16(8%) |  |

Pro: probiotics supplements

Pre: prebiotics supplements

Syn: synbiotics supplements

a: Chi-squared test

* Significant differences with P-value <0.05

**Supplementary Table 2.** Crude and adjusted ORs for HFMD related to probiotics, prebiotics, and synbiotics intakes.

|  | **Crude OR (95 % CI)** | **Adjusted OR (95 % CI)** | ***P* (Wald test)** |  |  |  |  |  |  |  | ***P* (LR-test)** |
| --- | --- | --- | --- | --- | --- | --- | --- | --- | --- | --- | --- |
| No supplements | Reference | Reference |  |  |  |  |  |  |  |  |  |
| Pre | 1.81 (0.65,5.06) | 1.65 (0.55,4.92) | 0.373 |  |  |  |  |  |  |  |  |
| Pro | 1.34 (0.93,1.93) | 1.59 (1.07,2.37) | 0.023^*^ |  |  |  |  |  |  |  |  |
| Syn | 0.39 (0.24,0.63) | 0.49 (0.29,0.82) | 0.007^*^ |  |  |  |  |  |  |  |  |
| Age | 0.53 (0.47,0.61) | 0.54 (0.47,0.62) | < 0.001^*^ |  |  |  |  |  |  |  | < 0.001^*^ |
| Sex (male) | 1.46 (1.08,1.99) | 1.35 (0.97,1.89) | 0.078 |  |  |  |  |  |  |  | 0.077 |

Pro: probiotics supplements

Pre: prebiotics supplements

Syn: synbiotics supplements

95 % CI: 95 % confidence interval

LR-test: likelihood ratio test

* Significant differences with P-value <0.05

**Supplementary Table 3.** Crude and adjusted ORs for HFMD related to probiotics, prebiotics, and synbiotics intakes in Propensity Score Matching (PSM) adjusted cohort.

|  | **OR (95 % CI)** | ***P* (Wald test)** |  |  |  |  |  |  |  |
| --- | --- | --- | --- | --- | --- | --- | --- | --- | --- |
| No supplements | Reference |  |  |  |  |  |  |  |  |
| Pre | 1.34 (0.39,4.65) | 0.646 |  |  |  |  |  |  |  |
| Pro | 1.8 (1.12,2.9) | 0.015^*^ |  |  |  |  |  |  |  |
| Syn | 0.45 (0.25,0.81) | 0.007^*^ |  |  |  |  |  |  |  |

Pro: probiotics supplements

Pre: prebiotics supplements

Syn: synbiotics supplements

95 % CI: 95 % confidence interval

* Significant differences with P-value <0.05
